# Seasonality and dynamics of schistosomiasis in the environment: usefulness of environmental DNA (eDNA) surveillance system at a community level for risk mapping schistosomiasis in Ekiran Village, Leyte, Philippines

**DOI:** 10.1128/msphere.01061-24

**Published:** 2025-03-26

**Authors:** Mark June Revolteado, Marcello Otake Sato, Joseph Valencia, Mario Jiz, Eleonor Cervantes, Ralph Aniceto, Marianette Inobaya, Darren Gray, Catherine A. Gordon, Pengfei Cai, Yasuhito Sako, Megumi Sato

**Affiliations:** 1 Graduate School of Health Sciences, Niigata University 594289 , Niigata, Niigata Prefecture, Japan; 2 Division of Global Environment Parasitology, Faculty of Medical Technology, Niigata University of Pharmacy and Medical and Life Sciences, Niigata, Niigata Prefecture, Japan; 3 Immunology Department, Research Institute for Tropical Medicine, Philippine Department of Health 433382 , Muntinlupa, Metro Manila, Philippines; 4 Department of Epidemiology and Biostatistics, Research Institute for Tropical Medicine, Philippine Department of Health, Muntilupa, Metro Manila, Philippines; 5 Global Health & Tropical Medicine, QIMR Berghofer Medical Research Institute, Brisbane, Queensland, Australia; 6 Applied Tropical and Molecular Parasitology Laboratory, QIMR Berghofer Medical Research Institute, Brisbane, Queensland, Australia; 7 Faculty of Medicine, University of Queensland, Brisbane, Queensland, Australia; 8 Molecular Parasitology Laboratory, QIMR Berghofer Medical Research Institute, Brisbane, Queensland, Australia; 9 Division of Parasitology, Department of Infectious Diseases, Asahikawa Medical University 38051 , Asahikawa, Hokkaido Prefecture, Japan; Virginia-Maryland College of Veterinary Medicine, Blacksburg, Virginia, USA

**Keywords:** schistosomiasis, environmental DNA (eDNA), *Oncomelania hupensis quadrasi*, *Schistosoma japonicum*, qPCR

## Abstract

Schistosomiasis, primarily caused by *Schistosoma japonicum* (Sj) in Asia, remains a major health concern in the Philippines, affecting 12.4 million people and causing symptoms like fever, abdominal pain, and hepatosplenomegaly. Chronic disease leads to stunting in children, and reinfection persists despite efforts to reduce morbidity. Current strategies focused on mass drug administration (MDA) and sporadic snail surveys, leaving gaps in monitoring and mitigating schistosomiasis transmission in the environment. To address these issues, this study refined an environmental DNA (eDNA)-based qualitative real-time polymerase chain reaction assay by making it field-applicable and multiplex, to detect both the parasite *S. japonicum* and its intermediate snail host, *Oncomelania hupensis quadrasi* (Ohq), using water samples. We surveyed the 30 sentinel sites quarterly—from July 2023 to March 2024—in Ekiran Village, Alangalang, Leyte, Philippines. Collectively, the eDNA of *O.h. quadrasi* was detected in 18 sites and that of *S. japonicum* eDNA was detected in 16 sites, while direct snail observation confirmed the presence in only five sites, with infected snails found in only one site. Consequently, the assay described temporal variation of Ohq and Sj, revealing the dynamics of Ohq colonies and Sj in Ekiran’s water sources. The eDNA confirmed the focality of Ohq and showed the erratic presence of Sj. Interestingly, both target species’ eDNA was observed more during the rainy season (December and March), which suggests a higher infection probability during this period. Integrating eDNA detection system with the existing control programs will enhance the identification of transmission hotspots, which may aid in reducing exposure risk for both humans and animals in the endemic areas.

**IMPORTANCE:**

This study aimed to fill the gaps in monitoring and mitigating schistosomiasis transmission in the environment. This field-applicable environmental DNA (eDNA)-based qualitative real-time polymerase chain reaction (qPCR) detection system focused on effectively detecting *Schistosoma japonicum* and its snail intermediate host, *Oncomelania hupensis quadrasi*, at the community level, moving from the traditional detection methods that are labor-intensive, less sensitive, and exposing surveyors to potential risk of infection. By introducing a field-applicable eDNA-based qPCR assay, this research provides a sensitive, non-invasive, and rapid molecular method for detecting *S. japonicum* and *O.h. quadrasi* in the environment. Additionally, the study not only provided insights in enhanced surveillance strategies but also contributed to a holistic eco-health approach by generating hazard maps for potential transmission and contamination sites, which could improve future control efforts and resource allocation for schistosomiasis elimination.

## INTRODUCTION

In the Philippines, schistosomiasis japonica, caused by the blood fluke *Schistosoma japonicum*, requires a specific freshwater snail intermediate host, *Oncomelania hupensis quadrasi*, to complete its life cycle and can infect numerous mammalian species including humans (1). Schistosomiasis remains a public health burden in the Philippines—causing acute systemic symptoms including fever, abdominal pain, diarrhea, hepatosplenomegaly, and growth stunting (1, 2)—impacting approximately 10% of the country’s population living in endemic areas with 20.83% of these people directly exposed to the parasite (1). Schistosomiasis control measures in the Philippines such as mass drug administration (MDA) using Praziquantel (PZQ), health education, improvement of sanitation, and snail monitoring and control are being carried out but not sustained (2). Additionally, traditional methods for detecting the parasite and its snail intermediate host in the environment typically require labor-intensive snail collection and microscopy (3), which are not only time-consuming but also less sensitive in low-prevalence settings (4). Given that, innovative approaches to environmental detection should be explored (3).

From an eco-health perspective, it is important to address not only the human factors but also the environmental and zoological (5) factors of the disease to effectively manage the spread and potentially eradicate the disease in the future (6). The current practice of snail monitoring in the Philippines is done by direct observation of the snails, in previously monitored areas and potential areas carried out by municipal malacologists (3, 7). However, mistakes in classifying the snails, especially those that closely resemble *O.h. quadrasi*, are inevitable. Additionally, this method directly exposes these people to the disease and, in turn, puts them at risk. Furthermore, human resources for this task are declining (7) as less attention is being given to (snail) vector control (5).

Recent advances in molecular biology have introduced environmental DNA (eDNA) as a non-invasive tool for monitoring biodiversity (8) and detecting infectious organisms (9) in various habitats. This method involves collecting genetic material directly from environmental samples, e.g., water, soil, and air, without the need to capture or visually identify the organisms (10). In the previous study conducted in the Philippines, the eDNA detection method was applied in parasitology, using the mitochondrial gene cytochrome c oxidase subunit I (COI) as a target for detection showing promising results, successfully detecting eDNA from *S. japonicum* and *O.h. quadrasi* from laboratory setup and actual field samples (11).

This study builds on these findings by developing and validating a qualitative real-time polymerase chain reaction (qPCR) assay that is both sensitive and rapid for detecting *S. japonicum* and *O.h. quadrasi* eDNA. Additionally, we aimed to assess the seasonality and dynamics of schistosomiasis at a community level in endemic areas using an improved field-applicable eDNA detection system for surveillance. A further goal was to create a hazard map of potential transmission and contamination sites based on the presence of *O.h. quadrasi* and *S. japonicum* eDNA throughout the year. This hazard map shall assist, if not enable, the local government unit to create effective control strategies and health policies in Ekiran Village Alangalang, Leyte (5).

## RESULTS

### Sampling sites, sampling periods, and water quality

**Fig 1:**
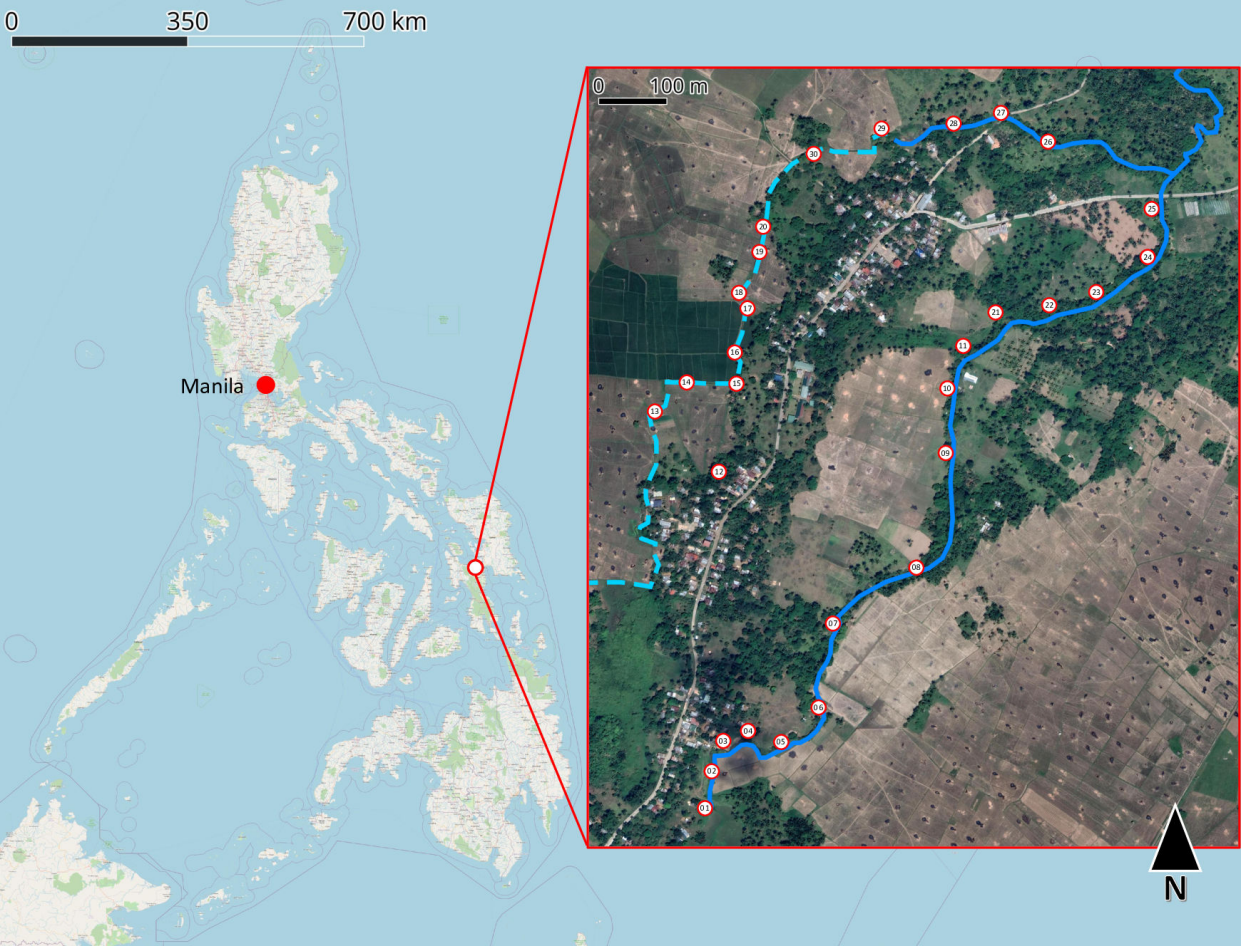
The Philippines and the detailed satellite map of Ekiran Village (and the 30 sampling points). This map is generated using QGIS ver. 3.32.3 Lima. The details of the map were enhanced to clearly depict the bodies of water sampled. The dashed line in lighter blue color represents the irrigation system that runs along the rice field of the village. This irrigation system is connected to the natural water stream that is represented by the continuous line in darker blue color. The white dots represent the points where the water samples were taken. This natural stream further extends to the Mainit river that runs along several municipalities in Leyte. Additionally, the natural stream is also used to supply water for the crops in the adjacent rice field.

This study was conducted in Ekiran Village, located in Alangalang, Leyte, where a long, continuous water system functions as a primary water source for both livelihood activities and as an irrigation system for the village’s rice fields (see Fig. 1). Given the focality of *O.h. quadrasi* population, a purposive sample collection was implemented and the sites were selected based on geographical features conducive to snail habitation (characterized by lush vegetation, water availability, and perpetual wetness), proximity to households with past or current schistosomiasis infections, and past reports of snail surveillance. This water system was divided into 30 sampling sites, spaced approximately 20–100 meters apart. The exact distances varied based on the site accessibility and the likelihood of human activity in the area. These criteria allowed us to focus on areas most likely to harbor both snail populations and potential schistosoma-contamination sites.

Field sampling was conducted four times—in July 2023, September 2023, December 2023, and March 2024—to assess whether periodic meteorologic changes impact eDNA detection. By sampling periodically, the study aimed to capture any variations in environmental conditions that could affect eDNA, thereby enhancing our understanding of how schistosomiasis detection may vary across periods. During each sampling event, we collected water samples and measured water quality parameters, including pH, temperature, conductivity, and total dissolved solids (TDS), using the Pocket Water Quality Meter HI-98129 (Combo 1, HANNA Instruments, USA). These measurements are critical in understanding the microenvironment of each site and how factors like the aforementioned parameters may influence the presence and viability of environmental DNA in the water. GPS data were also recorded for each sampling site using a Garmin GPSMAP 64 Series device (Garmin Ltd., Kansas, USA) to ensure precise geolocation of each sample collection point for future reference and data correlation.

The 30 sampling points can be categorized into two primary groups based on their role within the water system: irrigation system points and natural stream points (see Fig. 1). Although there are similarities in the general characteristics of sites within each group, distinct qualitative differences were noted in water quality and overall site conditions across individual sampling points (see Table 1). For example, some points had muddy, sediment-laden water, while others were clear and free-flowing. These variations were documented by measuring water quality parameters, presented in Tables 2–5. This table summarizes the data collected at each site, providing mean and standard deviation values both across all sampling periods and across all sampling sites within each period. Such detailed metrics allow for an in-depth analysis of water quality fluctuations over time and between different site conditions.

**TABLE 1:** General description of the sampling sites in Ekiran Village, Alangalang, Leyte

Site no	General site description
1	End of the natural water stream near the rice field, with presence of human waste, water is dirty and foul smelling
2	Natural stream adjacent to rice field
3	Natural stream adjacent to rice field and pig house
4	Natural stream adjacent to rice field, close to residents
5	Natural stream, carabaos were observed
6	Natural stream, carabaos were observed
7	Natural stream, carabaos were observed
8	Natural stream with lush vegetation
9	Rice paddy pump in natural stream
10	Natural stream adjacent to rice field, taro plantation
11	Natural stream, lush vegetation, taro plantation
12	Fish pond adjacent to rice field with presence of *Pomacea canaliculata* eggs
13	Irrigation canal, almost dry in December and March sampling
14	Irrigation canal, with presence of other snails
15	Irrigation canal, with presence of other snails
16	Part of the irrigation canal with pump, carabao wallow
17	Irrigation canal
18	Rice field adjacent to site 17
19	Irrigation canal adjacent to rice field
20	Irrigation canal adjacent to rice field
21	Natural stream with muddy surrounding and lush vegetation
22	Natural stream with lush vegetation
23	Natural stream with lush vegetation
24	Natural stream with bamboo bridge, lush vegetation present, carabaos are tied in the water
25	Natural stream with lush vegetation
26	Natural stream with lush vegetation
27	Natural stream with lush vegetation, carabaos are tied in the water
28	Natural stream with lush vegetation
29	Part of a natural stream with lush vegetation connected to irrigation
30	Part of the irrigation canal, deep carabao wallow

To provide specific insights into water quality parameters, Table 2 displays the pH levels recorded at each sample site across the sampling periods. Table 3 shows the recorded water temperatures (in degrees Celsius; °C), Table 4 provides water conductivity (measured in milliSiemens per centimeter; mS), and Table 5 presents the total dissolved solids (TDS) in the water, measured in parts per trillion (ppt). These water quality metrics offer valuable information regarding the physicochemical properties of each sampling site and how these factors might impact the persistence and detectability of eDNA. During the March 2024 sampling, no samples were collected from site number 18, as the rice field was dry, resulting in no data.

**TABLE 2:** Water pH by collection site across sampling periods in Ekiran Village, Alangalang, Leyte, Philippines (2023–2024)

Site number	July 2023	September 2023	December 2023	March 2024	Mean (per site)	SD (per site)
1	6.90	6.84	7.07	6.86	6.92	0.09
2	7.00	6.93	6.98	7.47	7.10	0.22
3	7.16	7.15	7.15	7.41	7.22	0.11
4	7.01	7.02	6.96	7.35	7.09	0.15
5	7.12	6.95	6.89	7.33	7.07	0.17
6	7.14	6.77	7.28	6.94	7.03	0.19
7	7.08	6.93	6.90	7.24	7.04	0.14
8	6.97	6.85	6.89	6.93	6.91	0.04
9	7.00	7.05	6.94	7.12	7.03	0.07
10	6.71	7.11	7.41	7.45	7.17	0.30
11	7.01	7.25	7.08	7.28	7.16	0.11
12	7.10	6.44	6.64	7.25	6.86	0.33
13	7.13	7.31	6.77	7.22	7.11	0.20
14	7.10	7.21	7.10	7.09	7.13	0.05
15	7.10	6.98	6.97	7.33	7.10	0.15
16	7.10	7.01	6.92	7.02	7.01	0.06
17	7.15	7.03	7.12	7.31	7.15	0.10
18	9.30	7.09	7.08	–	7.82	1.04
19	7.07	7.13	7.08	7.05	7.08	0.03
20	7.31	7.15	7.30	7.04	7.20	0.11
21	6.86	7.28	7.11	7.29	7.14	0.17
22	7.14	7.26	7.11	7.47	7.25	0.14
23	7.08	7.28	6.98	7.41	7.19	0.17
24	7.13	7.18	7.04	7.28	7.16	0.09
25	7.29	7.38	7.24	7.32	7.31	0.05
26	6.96	7.42	7.14	7.32	7.21	0.18
27	7.03	7.27	7.00	7.32	7.16	0.14
28	7.01	7.18	7.30	7.44	7.23	0.16
29	7.00	7.18	7.12	7.32	7.16	0.12
30	7.04	7.16	7.16	7.29	7.16	0.09
Mean (per sampling)	7.13	7.09	7.06	7.25		
SD (per sampling)	0.42	0.20	0.16	0.17		

^*a*^ – indicates no detection.

**TABLE 3:** Water temperature (°C) by collection site across sampling periods in Ekiran Village, Alangalang, Leyte, Philippines (2023–2024)

Site number	July 2023	September 2023	December 2023	March 2024	Mean (per site)	SD (per site)
1	28.60	31.70	27.90	26.30	28.63	1.96
2	28.90	33.10	27.70	26.00	28.93	2.62
3	29.40	33.90	27.70	26.50	29.37	2.81
4	29.70	34.50	27.70	26.90	29.70	2.95
5	28.90	32.40	27.40	26.80	28.87	2.17
6	28.80	31.40	27.60	26.50	28.58	1.82
7	28.40	30.80	27.50	26.10	28.20	1.71
8	27.90	31.40	27.90	26.60	28.45	1.76
9	27.90	30.40	27.70	25.90	27.98	1.60
10	29.00	30.00	27.80	25.70	28.13	1.53
11	27.50	28.80	27.70	25.70	27.43	1.11
12	29.80	29.80	26.90	26.40	28.23	1.32
13	32.30	30.10	28.70	27.40	29.63	1.03
14	32.10	29.80	27.90	29.50	29.83	0.79
15	31.60	29.90	27.00	30.50	29.75	1.35
16	32.00	30.80	28.00	27.10	29.48	1.41
17	32.90	31.40	27.80	29.80	30.48	1.32
18	38.20	31.30	32.50	–	34.00	1.10
19	33.00	32.10	28.60	30.00	30.93	1.28
20	34.90	32.20	31.00	32.50	32.65	0.65
21	26.60	29.00	27.60	26.20	27.35	1.00
22	26.50	29.10	27.30	25.70	27.15	1.21
23	26.60	29.00	27.30	25.70	27.15	1.17
24	26.40	29.10	27.30	26.00	27.20	1.11
25	26.40	29.20	27.70	25.80	27.28	1.21
26	28.30	29.50	28.00	26.60	28.10	1.03
27	29.00	30.70	29.30	27.50	29.13	1.13
28	28.40	30.20	27.50	27.50	28.40	1.10
29	29.90	29.20	27.60	26.50	28.30	0.99
30	29.70	30.60	27.80	28.20	29.08	1.07
Mean (per sampling)	29.76	30.71	28.01	27.17		
SD (per sampling)	2.91	1.46	1.12	1.68		

^*a*^ – indicates no detection.

**TABLE 4:** Water conductivity (mS) by collection site across sampling periods in Ekiran Village, Alangalang, Leyte, Philippines (2023–2024)

Site number	July 2023	September 2023	December 2023	March 2024	Mean (per site)	SD (per site)
1	0.20	0.18	0.16	0.48	0.26	0.13
2	0.22	0.28	0.22	0.21	0.23	0.03
3	0.22	0.29	0.22	0.22	0.24	0.03
4	0.22	0.29	0.22	0.23	0.24	0.03
5	0.23	0.32	0.22	0.21	0.25	0.04
6	0.11	0.34	0.23	0.26	0.24	0.08
7	0.11	0.37	0.23	0.26	0.24	0.09
8	0.12	0.37	0.26	0.27	0.26	0.09
9	0.12	0.37	0.25	0.28	0.26	0.09
10	0.08	0.38	0.25	0.28	0.25	0.11
11	0.12	0.38	0.25	0.27	0.26	0.09
12	0.16	0.12	0.21	0.14	0.16	0.03
13	0.23	0.22	0.19	0.19	0.21	0.02
14	0.23	0.22	0.14	0.20	0.20	0.03
15	0.23	0.21	0.14	0.20	0.20	0.03
16	0.23	0.20	0.14	0.17	0.19	0.03
17	0.23	0.20	0.14	0.18	0.19	0.03
18	0.22	0.20	0.17	–	0.20	0.02
19	0.24	0.22	0.15	0.25	0.22	0.04
20	0.26	0.22	0.27	0.28	0.26	0.02
21	0.12	0.38	0.25	0.28	0.26	0.09
22	0.12	0.38	0.25	0.28	0.26	0.09
23	0.12	0.40	0.27	0.29	0.27	0.10
24	0.14	0.44	0.27	0.30	0.29	0.11
25	0.14	0.45	0.22	0.32	0.28	0.12
26	0.17	0.28	0.21	0.38	0.26	0.08
27	0.12	0.29	0.22	0.35	0.25	0.09
28	0.16	0.27	0.19	0.33	0.24	0.07
29	0.12	0.24	0.19	0.31	0.22	0.07
30	0.16	0.24	0.18	0.31	0.22	0.06
Mean (per sampling)	0.17	0.29	0.21	0.27		
SD (per sampling)	0.05	0.08	0.04	0.07		

^*a*^ – indicates no detection.

**TABLE 5:** Total dissolved solids (ppt) in water by collection site across sampling periods in Ekiran Village, Alangalang, Leyte, Philippines (2023–2024)

Site number	July 2023	September 2023	December 2023	March 2024	Mean (per site)	SD (per site)
1	0.11	0.09	0.08	0.24	0.13	0.06
2	0.11	0.14	0.11	0.10	0.12	0.01
3	0.11	0.15	0.11	0.11	0.12	0.02
4	0.11	0.15	0.11	0.12	0.12	0.01
5	0.11	0.16	0.11	0.11	0.12	0.02
6	0.05	0.17	0.11	0.13	0.12	0.02
7	0.06	0.19	0.12	0.13	0.13	0.03
8	0.06	0.18	0.13	0.14	0.13	0.02
9	0.06	0.18	0.13	0.14	0.13	0.02
10	0.04	0.19	0.13	0.14	0.13	0.03
11	0.06	0.19	0.12	0.14	0.13	0.03
12	0.08	0.06	0.12	0.07	0.08	0.02
13	0.12	0.11	0.09	0.10	0.11	0.01
14	0.11	0.11	0.07	0.10	0.10	0.01
15	0.11	0.10	0.07	0.10	0.10	0.01
16	0.11	0.10	0.07	0.09	0.09	0.01
17	0.11	0.10	0.07	0.09	0.09	0.01
18	0.11	0.10	0.09	–	0.10	0.00
19	0.12	0.11	0.08	0.13	0.11	0.02
20	0.13	0.11	0.13	0.14	0.13	0.01
21	0.06	0.19	0.13	0.14	0.13	0.02
22	0.06	0.19	0.12	0.14	0.13	0.03
23	0.06	0.20	0.13	0.14	0.13	0.03
24	0.07	0.22	0.13	0.15	0.14	0.04
25	0.07	0.24	0.11	0.16	0.15	0.05
26	0.06	0.14	0.11	0.19	0.13	0.03
27	0.06	0.14	0.11	0.18	0.12	0.03
28	0.08	0.13	0.10	0.17	0.12	0.03
29	0.06	0.12	0.10	0.15	0.11	0.02
30	0.08	0.12	0.09	0.15	0.11	0.02
Mean (per sampling)	0.08	0.15	0.11	0.13		
SD (per sampling)	0.03	0.04	0.02	0.03		

^*a*^ – indicates no detection.

Within Ekiran Village, parts of the natural stream run adjacent to rice fields (see Fig. 1), providing a water source that sustains the crops during dry periods. Observations made at each sampling point revealed that, in many of these areas, carabaos were often present (Table 1). According to village residents, carabaos are typically leashed in these locations when not being used for agricultural work. The presence of dense vegetation, combined with constant access to water and natural shade, provides a favorable environment for carabaos to rest and avoid the intense tropical heat. However, this proximity of carabaos to the water system is of particular concern, as these animals are known definitive hosts of *Schistosoma japonicum*, the parasite responsible for schistosomiasis.

### Comparison of eDNA detection system by qPCR and traditional (direct observation) method

The qPCR analysis for both targets was conducted in triplicates, with the reported Cq values in Tables 6 and 7 representing the mean Cq for each site. This approach ensured reproducibility and consistency in the Cq measurements. Additionally, the number of snails collected per site was recorded and analyzed alongside the qPCR results for the Ohq-COI target.

**TABLE 6:** Detection of *O.h. quadrasi* in Alangalang, Leyte, Philippines, in 2023–2024 by sampling sites^*a*^,^*b*^

Sampling site no.	JULY 2023		September 2023		December 2023		March 2024	
	qPCR (Cq)	Traditional	qPCR (Cq)	Traditional	qPCR (Cq)	Traditional	qPCR (Cq)	Traditional
1	–	6	–	4	36.30	10	–	3
2	–	–	–	–	–	–	38.12	–
4	–	–	–	–	–	–	37.69	–
6	–	1	36.52	–	38.03	–	38.19	–
7	–	–	–	–	–	2	39.15	–
9	–	–	36.45	–	37.79	–	36.65	–
10	–	19	35.58	3	36.52	5	34.33	13
11	36.76	–	41.98	–	–	–	39.09	–
12	–	–	–	–	36.97	–	–	–
13	38.34	–	–	–	36.48	–	–	–
14	–	–	37.49	–	38.47	–	39.23	–
15	–	–	36.36	–	37.96	–	–	–
19	–	–	37.53	–	–	–	–	–
21	38.84	20	–	–	–	10	–	1
22	38.59	–	–	–	–	–	–	–
23	38.19	–	–	–	–	–	–	–
27	38.93	–	–	–	37.64	–	–	–
29	–	–	38.25	–	–	–	–	–
Positivity (*n*)	6	4	8	2	9	4	8	3
Positivity rate	20.00%	13.33%	26.67%	6.67%	30.00%	13.33%	27.59%	10.34%
Agreement, *n* (%)	22 (73.33%)		22 (73.33%)		21 (70.00%)		20 (68.97%)	

^*a*^ Comparison of detection rate between qPCR (Ohq-COI target) results and number of the collected *O.h. quadrasi* snails.

^*b*^ – indicates no detection.

**TABLE 7:** Detection of *S. japonicum* in Alangalang, Leyte, Philippines, in 2023–2024 by sampling sites^*a*^

Sampling site no.	JULY 2023		September 2023		December 2023		March 2024	
	qPCR (Cq)	Traditional	qPCR (Cq)	Traditional	qPCR (Cq)	Traditional	qPCR (Cq)	Traditional
1	–	1	–	–	38.88	1	–	–
8	–	–	–	–	–	–	38.45	–
9	–	–	–	–	–	–	32.86	–
11	–	–	–	–	–	–	37.27	–
12	–	–	–	–	–	–	38.74	–
13	–	–	35.38	–	–	–	–	–
17	–	–	–	–	42.98	–	–	–
20	39.02	–	–	–	–	–	–	–
21	–	–	–	–	–	–	38.14	–
22	–	–	–	–	38.62	–	38.34	–
23	–	–	–	–	–	–	29.69	–
24	–	–	38.87	–	–	–	–	–
25	–	–	–	–	–	–	36.21	–
26	39.05	–	–	–	–	–	–	–
29	39.44	–	–	–	–	–	–	–
30	–	–	–	–	–	–	39.26	–
Positivity (*n*)	3	1	2	0	3	1	9	0
Positivity rate	10.00%	3.33%	6.67%	0.00%	10.00%	3.33%	31.03%	0.00%
Agreement, *n* (%)	26 (86.67%)		28 (93.33%)		28 (93.33%)		20 (68.97%)	

^*a*^ Comparison of detection rate between qPCR (Sj-COI target) results and number of the collected *O.h. quadrasi* snails infected with *S. japonicum.*

^*b*^ – indicates no detection.

In terms of *S. japonicum* presence detection, snails collected from each site were crushed and examined through microscopy. The sites were considered *S. japonicum* positive for the traditional method if sporocysts or furcocercous bifurcated cercariae were observed, providing a direct visual confirmation of infection. These microscopy results were subsequently compared to the qPCR (Sj-COI target) findings to evaluate the sensitivity and specificity of the qPCR method relative to traditional snail-crushing and microscopy. The Wilcoxon signed-rank test was applied to assess the statistical significance between qPCR and direct observation methods, with *P* values ≤ 0.05 indicating a statistically significant difference between these two detection techniques.

During the initial collection in July 2023, *O.h. quadrasi* was observed in four sites through traditional methods, while qPCR detected positivity in six sites. This discrepancy suggests that qPCR may have detected lower concentrations of *O.h. quadrasi* DNA that the traditional method might have missed. Similarly, in September 2023, traditional methods found *O.h. quadrasi* in only two sites, while qPCR identified eight positive sites, further highlighting qPCR’s potential for higher sensitivity. By December 2023, the traditional method noted *O.h. quadrasi* in four sites, while qPCR detected positivity in nine sites. Finally, in March 2024, *O.h. quadrasi* was observed in four sites and was detected in eight sites by qPCR (Table 6).

Although variability existed in detection rates between traditional methods and qPCR across survey periods, the overall percent agreement between the two methods was high, averaging 71.41%. This substantial agreement indicates that qPCR is more sensitive—with a detection limit of 1 copy/5 µL of the target COI mtDNA (Supplemental material)—yet moderately concordant with the traditional method. Across 30 sites surveyed, qPCR identified a mean positivity rate of 26.07%, whereas the traditional method had a lower average positivity rate of only 10.91%. Collectively, qPCR detected *O.h. quadrasi* positivity in 18 sites throughout the survey period, compared to only five sites detected by the traditional method. These findings strongly suggest that qPCR is generally more effective at detecting *O.h. quadrasi* presence over multiple survey periods.

For the detection of *S. japonicum*, similar patterns emerged across survey periods.(Table 7) During the initial collection in July 2023, infected *O.h. quadrasi* snails were observed at one site, while qPCR detected Sj positivity at three sites. This discrepancy in July highlights qPCR’s ability to detect DNA from *S. japonicum* even when snail population might be too subtle for direct observation. In September 2023, no infected snails were found using traditional methods, while qPCR identified Sj positivity at two sites. In December 2023, infected snails were observed at one site, whereas qPCR detected Sj positivity at three sites. By March 2024, no infected *O.h. quadrasi* was detected through traditional observation, yet qPCR found Sj positivity at nine sites. This increase in qPCR-detected positivity across sampling timepoints supports its sensitivity for capturing low-prevalence areas.

Throughout the survey periods, the traditional and qPCR methods showed a high overall percent agreement of 85.58% in detecting *S. japonicum*. Despite this agreement, qPCR demonstrated a significantly higher sensitivity—with a detection limit of 1 copy/5 µL of the target COI mtDNA (Supplemental material)—capturing more instances of *S. japonicum* positivity than traditional snail-crushing and microscopy. Of the 30 sites surveyed, qPCR had an average positivity rate of 14.43%, while the traditional method yielded a considerably lower rate of 1.67%. Over the entire sampling period, the eDNA detection system by qPCR found *S. japonicum* positivity in 16 sites, in contrast to only one site confirmed through traditional methods. These findings highlight qPCR’s robustness in detecting *S. japonicum*, even when visual confirmation through snail collection and crushing might not yield reliable signs of environmental presence.

Across multiple survey periods, the number of positive sites detected by either traditional methods or qPCR varied significantly, as indicated by the collective statistically significant difference in detecting *O.h. quadrasi* and *S. japonicum*. However, this difference was not observed in all the sampling timepoints as presented in Table 8. In detecting *O.h. quadrasi* in the environment, the two methods significantly varied only during the September sampling. Moreover, the detection of *S. japonicum* between the two methods only showed a significant difference during the last sample collection in March 2024. Regardless, the detection of environmental presence of both target species by qPCR was higher compared to traditional methods in all sampling periods.

**TABLE 8:** Comparison between the performance of *O.h. quadrasi* collection and microscopy and qPCR using the Wilcoxon signed-rank test

Target species	July 2023 (*P*)	September 2023 (*P*)	December 2023 (*P*)	March 2024 (*P*)	TOTAL (*P*)
*O.h. quadrasi*	0.480	0.034^*a*^	0.096	0.096	0.002^*a*^
*S. japonicum*	0.317	0.157	0.157	0.003^*a*^	<0.001^*a*^

^*a*^ Statistically significant difference.

### Environmental DNA positivity and water quality

To assess whether water quality affects the detection of *S. japonicum* and *O.h. quadrasi* eDNA, we subjected the water parameters collected—pH, temperature, conductivity, and TDS—to statistical analysis. The Mann-Whitney *U* test was used to determine if there is a significant difference in water quality measures between sites with and without the presence of *S. japonicum* and *O.h. quadrasi* eDNA for the total sampling conducted. A *P* value ≤ 0.05 indicated a statistically significant difference between qPCR positive and qPCR negative sites.

**Fig 2:**
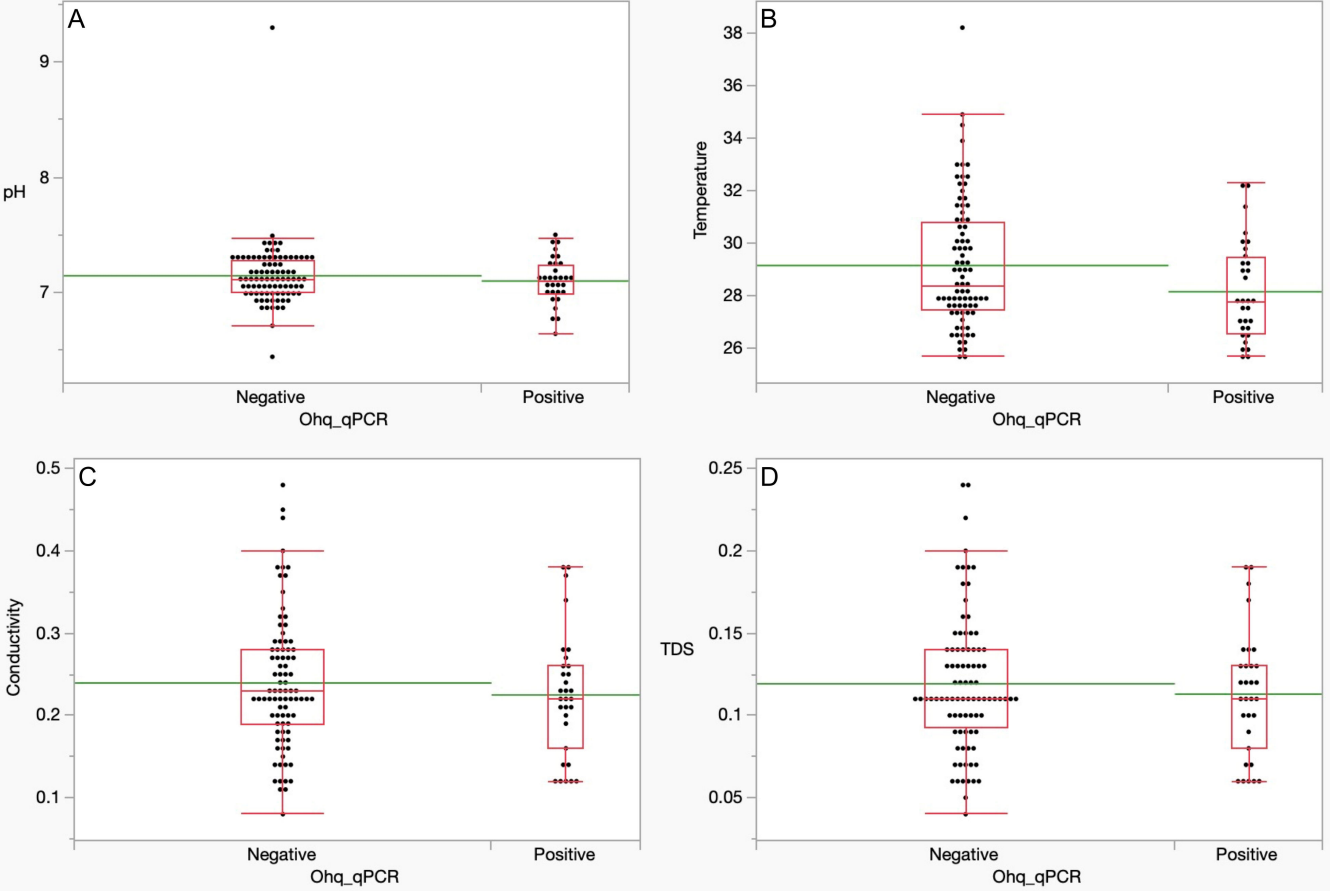
Boxplot of Mann-Whitney *U* analysis of water quality of sampling sites according to qPCR positivity for the Ohq-COI target. The green line represents the mean for each graph. (A) pH, (B) temperature, (C) conductivity, and (D) total dissolved solids.

The Mann-Whitney *U* test revealed that there is no statistically significant difference (*P* > 0.05) between sites with the presence of *O.h. quadrasi* eDNA and sites without *O.h. quadrasi* eDNA across all water parameters examined. This indicates that the water quality measurements do not significantly differ between the Ohq eDNA positive and Ohq eDNA negative sites. However, the means of all water parameters for sites that tested positive for *O.h. quadrasi* are lower compared to those areas that tested negative by qPCR(Table 9; Fig. 2).

**TABLE 9:** Mann-Whitney *U* analysis of different water quality of sampling sites according to qPCR positivity for Ohq-COI target

Parameter	qPCR positive mean rank	qPCR negative mean rank	S	Z	*P*
pH	56.55	61.22	1753	−0.645	0.519
Temperature	48.03	61.04	1,489	−1.867	0.062
Conductivity	55.35	60.98	1,716	−0.784	0.433
TDS	56.95	61.07	1,765.5	−0.573	0.567

**Fig 3:**
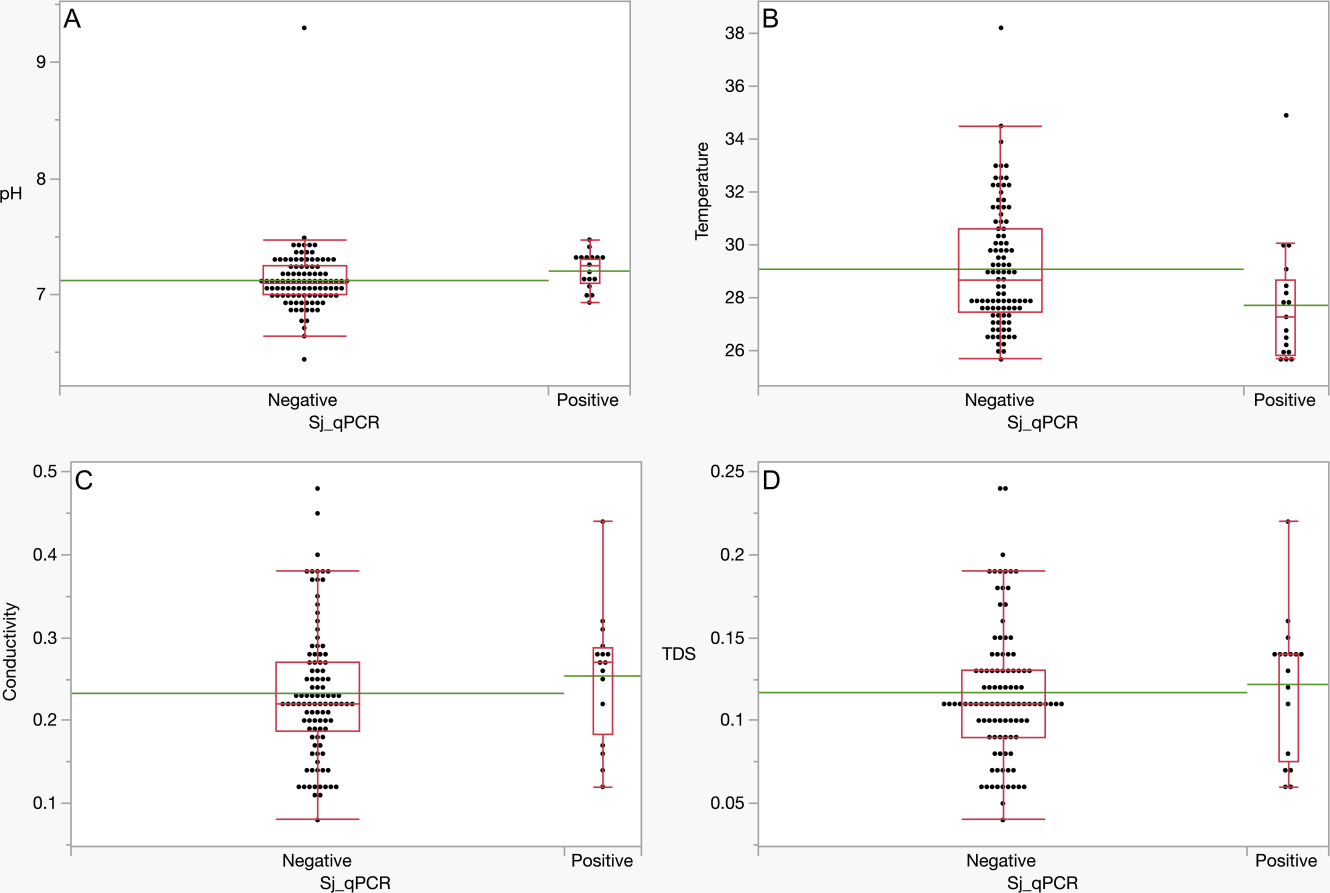
Boxplot of Mann-Whitney *U* analysis of water quality of sampling sites according to qPCR positivity for the Sj-COI target. The green line represents the mean for each graph. (A) pH, (B) temperature, (C) conductivity, and (D) total dissolved solids.

In contrast to the result of Ohq qPCR and water quality, the test revealed a statistically significant difference in water pH and temperature (*P* < 0.05) between sites with the presence of *S. japonicum* eDNA and sites without *S. japonicum* eDNA. Specifically, the mean values suggest that bodies of water with a pH 7.2 ± 0.16 and a lower temperature of approximately 27.74 ± 2.36°C tend to harbor *S. japonicum*. Conversely, no statistically significant difference was found for water conductivity and TDS (*P* > 0.05). However, the means for sites where *S. japonicum* eDNA was detected are higher compared to sites where no Sj eDNA was detected. (Table 10; Fig. 3).

**TABLE 10:** Mann-Whitney *U* analysis of different water quality of sampling sites according to qPCR positivity for Sj-COI target

Parameter	qPCR positive mean rank	qPCR negative mean rank	S	Z	*P*
pH	75.65	57.39	1,286	2.017	0.044^*a*^
Temperature	38.53	60.82	655	−2.562	0.010^*a*^
Conductivity	70.84	57.72	1,133.5	1.426	0.154
TDS	67.62	58.73	1,149.5	0.986	0.324

^*a*^ Statistically significant difference.

### *O.h. quadrasi* and *S. japonicum* temporal distribution map across sampling periods

After analyzing the samples to detect both target species—*S*. *japonicum* and *O.h. quadrasi—*a hazard map was generated for each sampling period. This map plotting aimed to describe the temporal movement and distribution of *S. japonicum* and *O.h. quadrasi* within the environment by tracking the presence of their environmental DNA (eDNA). By mapping the eDNA presence of both target species, the study sought to understand how periodic meteorologic changes might influence the spread of *S. japonicum* and *O.h. quadrasi*. This approach allowed for a comprehensive assessment of environmental risks that may guide management strategies for these species

**Fig 4:**
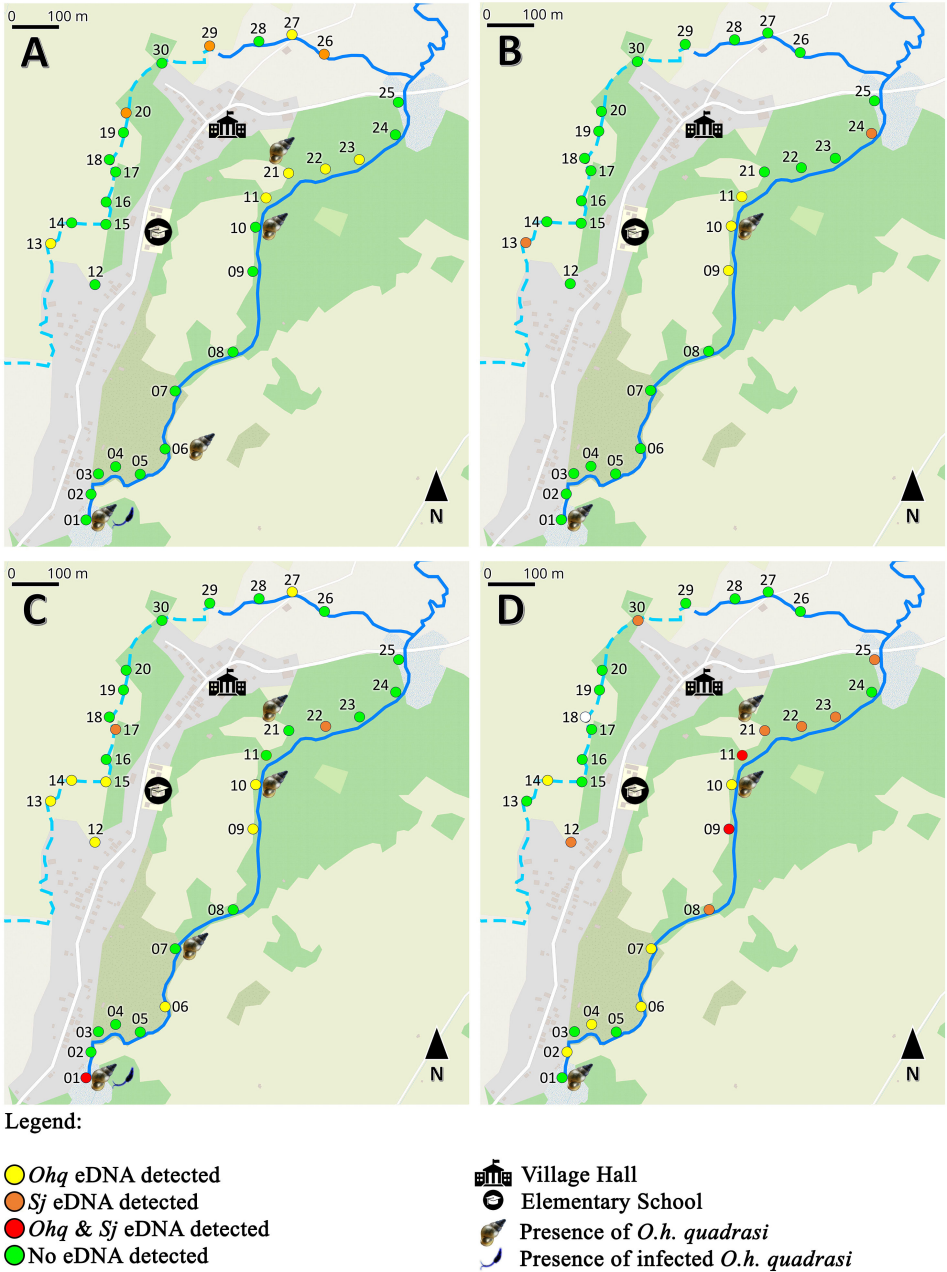
Generated hazard map according to the qPCR results of both target species and the malacological survey data for each sampling period using Google street map format, QGIS ver. 3.32.3 Lima. The details of the map were enhanced to clearly depict the bodies of water sampled. Similar to Fig. 1, the bodies of water sampled are represented in two distinct blue lines. The white line (branching in three parts toward the north part of the map) is the main road of the village. The gray area of the map shows the mixed-use area that includes the residential houses (represented in beige polygons), the village hall, and the elementary school. (A) July 2023 sampling period; (B) September 2023 sampling period; (C) December 2023 sampling period; and (D) March 2024 sampling period.

Four hazard maps were generated for each sampling period, illustrating the spatial and temporal distributions of the two target species (*O.h. quadrasi* and *S. japonicum*) based on eDNA detection by qPCR and traditional methods across various sampling points from July 2023 to March 2024. Each map represents a survey conducted in different months, with points color-coded to indicate the presence or absence of eDNA for both target species. For context, site 12 is not directly part of the irrigation system—being a separate fishpond—however, it has contact with water from the irrigation system as it is adjacent to a rice field that receives irrigation water. In Fig. 4, the village hall and elementary school served as reference points.

In the initial survey conducted in July 2023 (Fig. 4A), the majority of sampling points (21 out of 30) showed no detection of eDNA (green points). However, six of the 30 sites, particularly southeast of the village hall (sites 11 and 21 to 23), northwest of the village hall (site 27), and west of the elementary school (site 13), showed the presence of *O.h. quadrasi* eDNA (yellow points). Among these sites, *O.h. quadrasi* was observed only at site 21, where 20 individuals were collected. However, non-detection of *O.h. quadrasi* eDNA was noted at sites 6 and 10. *S. japonicum* eDNA (orange points) was detected at three scattered sites (20, 29, and 26), none of which had observable *O.h. quadrasi*, making detection of *S. japonicum* by traditional methods not feasible. In terms of eDNA positivity, no site showed co-detection of both species (red points). However, traditional methods revealed that one of the six *O.h. quadrasi* snails collected from site 1 was infected with *S. japonicum*.

In the second survey conducted in September 2023 (Fig. 4B), *O.h. quadrasi* eDNA positivity revealed a movement from the southeast side of the village hall. Positivity at sites 11 and 21 to 23 shifted to sites 9 to 11, consistent with malacological data showing the presence of *O.h. quadrasi* at site 10. Non-detection at site 1 persisted, with site conditions remaining a possible confounder. Interestingly, site 13, which previously tested positive for *O.h. quadrasi* eDNA, tested positive for *S. japonicum* eDNA in this survey. This likely reflects environmental contamination since no snails were collected and *O.h. quadrasi* eDNA was not detected at this site. Site 24, which also tested positive for *S. japonicum* eDNA, may similarly reflect environmental contamination, as carabaos were observed in that area (Table 1). No co-detection of either species was observed in September, either by eDNA qPCR or traditional methods.

In the third survey conducted in December 2023 (Fig. 4C), a spread of *O.h. quadrasi* eDNA was observed from site 12 to site 15, expanding from the previous detection at site 13 in the July survey. *S. japonicum* eDNA was detected at two sites, 17 and 22. Since no snails were collected at these sites, environmental contamination by animals and/or humans is likely. Non-detection of snail eDNA was observed in sites 7 and 21 where 2 and 10 snails were respectively found (see Table 6). Interestingly, eDNA detection in site 10 remained consistent with the malacological survey. Similarly in site 1, the detection of both target species by eDNA aligned with the malacological survey and microscopy. Site 9 remained positive for snail eDNA from the September 2023 survey, which was interesting as its adjacent site (site 10) has the presence of actual snails throughout the sampling periods. Additionally, snail eDNA positivity was observed again in site 27 in comparison with its detection during the July survey and non-detection during the September survey.

In the last survey conducted in March 2024 (Fig. 4D), no samples were collected at site 18 due to the rice field being dried at the time; hence, the point marking the site is indicated in white. Site 10 remained consistent with the malacological data, while non-detection at sites 1 and 21 persisted. Interestingly, co-detection of both target species was observed in sites 9 and 11 in which the eDNA of the snail was detected from the previous surveys. Consequently, in the vicinity (sites 8–10 and sites 21–25) of both sites, Sj eDNA positivity was also present. Another interesting observation was the detection of snail eDNA in sites 6 and 7 where no eDNA was previously detected but with observed presence of one and two snails in July and December survey, respectively.

Overall, the maps reveal distinct temporal and spatial patterns in the distribution of *O.h. quadrasi* and *S. japonicum* eDNA. *O.h. quadrasi* consistently appeared in the natural stream (as detected by both the eDNA detection system and traditional methods) across the survey period, indicating that this area provides a suitable habitat. The detection of *O.h. quadrasi* eDNA indicates that the distribution of *O.h. quadrasi* is focal—shifting temporally but not moving far from its previous locations—and remains concentrated where snail colonies were found. In contrast, *S. japonicum* eDNA detection exhibited greater fluctuation in both presence and distribution, with peaks observed in September and March.

## DISCUSSION

In a previous study, the specificity of the eDNA detection system was evaluated by testing primer designs for detecting *O.h. quadrasi* and *S. japonicum* against non-target snail species and trematodes, respectively, using conventional PCR and probe-based qPCR assays (11). The assay was further validated through a laboratory setup, where aquaria containing Sj-infected *O.h. quadrasi* were prepared as spiked samples. The experiment yielded promising results, as the eDNA detection system successfully amplified Ohq and Sj DNA samples (11).

In this study, we utilized the previously developed detection system (11, 12), with few technical modifications, and applied it as an actual monitoring tool at the village level. Especially, the modifications made in the probe yielded a higher sensitivity, being able to detect as low as 1 copy/5 µL of the target COI mtDNA of both target species in the laboratory setting (Supplemental material). This community-wide eDNA surveillance was conducted quarterly to assess and map the temporal and spatial distributions of *Oncomelania hupensis quadrasi* and *Schistosoma japonicum* in the environment of Ekiran Village, Alangalang, Leyte, Philippines, and to provide a broader perspective on the parasite and its hosts' potential impact on schistosomiasis incidence in the village.

The results highlighted the temporality of both *O.h. quadrasi* and *S. japonicum* DNA distributions over one year in an endemic area for *S. japonicum* in the Philippines. This variability may be due to differences in meteorological conditions, as observed in the increased positivity for both target species during the December and March collections, which correspond to periods of higher rainfall and lower temperatures (13).

Non-detection of eDNA at sites where Ohq was physically observed, however, raises important considerations regarding environmental factors and the snail intermediate host’s behavioral factors that may affect eDNA persistence (14) and detectability. For example, in the July survey at site 6, only one individual of *O.h. quadrasi* was collected, and subsequent surveys revealed no presence of the snail in that location. It is likely that the encounter at site 6 was accidental (15)—considering frequent human and animal activities in this area—which may explain the absence of detectable eDNA. In the case of site 10, water quality may have been a confounding factor for non-detection (10, 14). As shown in Table 9, although not statistically significant, the mean for water quality parameters (pH, temperature, conductivity, and TDS) in sites that tested negative for *O.h. quadrasi* eDNA was generally higher than those in sites that tested positive. Specifically, during the collection at site 10, pH, conductivity, and TDS were at their lowest compared to other sampling periods. Additionally, in later surveys, the detection of *O.h. quadrasi* eDNA aligned with the malacological data for site 10. On the other hand, several factors could explain the non-detection of either target species at site 1 in the July survey by qPCR, including the presence of contaminants such as garbage and the overall condition of the area, as described in Table 1. These factors may have interfered with the quality of the eDNA collected from this site (10, 14).

During the December survey, a similar explanation to that of site 6 in the July survey may apply to the observation at site 7, as only two individuals of *O.h. quadrasi* were collected during the survey, and they were only observed at that time. A potential mechanism of accidental transfer could be the snail's attachment to tools and equipment carried by humans or to their clothes or footwear, as well as animals' skin (15). In the case of site 21, although 10 individuals of *O.h. quadrasi* were collected and had previously been detected by qPCR, during this sample collection, the snails were collected from the surrounding soil and vegetation rather than from the body of water (16, 17), which may explain the absence of detectable eDNA in the water samples. Also in December, co-detection of both *O.h. quadrasi* and *S. japonicum* was observed at site 1, and a possible reason for detectability may have been the water conditions (10, 14) at the time of sampling. Specifically, the pH and temperature at that time were optimal for both *O.h. quadrasi* and *S. japonicum* (16–18), as presented in the measured water parameters (see Tables 2 and 3).

During the last survey conducted in March 2024, non-detection in sites 1 and 21 persisted. In the case of site 1, only during the December survey did the water quality change in a way that may have favored the limitations of eDNA expression from both target species (10, 14, 19). In the case of site 21, only one individual of *O.h. quadrasi* was collected. In addition, although it was not measured, the water level during the collection was visibly lower compared to previous sampling periods. This might have impacted the snail population during that period (17, 18), thereby affecting the eDNA presence.

The amphibious behavior of *O.h. quadrasi* may influence the amount of DNA released into the water by reducing direct shedding or causing intermittent eDNA deposition (15–18). Additionally, burrowing of adult snails into the soil during the hottest hours of the day to avoid desiccation (12) makes them visually undetectable, and the detection of *O.h. quadrasi* eDNA in locations where no snails were visually observed could be attributed to the presence of juvenile snails or snail eggs. Both juvenile and eggs of *O.h. quadrasi* are very small (<0.5 mm) and difficult to detect with the naked eye in natural settings (16–18).

Environmental factors such as water temperature, pH, microbial activity, and the presence of natural or anthropogenic contaminants can contribute to eDNA degradation or dilution, increasing the likelihood of false negatives (10, 12, 14). These challenges underscore the need for further refinement of eDNA protocols, particularly in complex or dynamic environments.

Improvements in sample collection strategies, such as optimizing the timing and frequency of water sampling to account for snail behavioral patterns (15–18), could enhance detection efficiency. Environmental DNA analysis in aquatic ecosystems can be significantly hindered by natural inhibitors such as humic substances, fine sediments, and algal matter (20). These inhibitors interfere with polymerase chain reaction (PCR) amplification, leading to false negatives or an underestimation of target DNA concentrations. For instance, humic acids, prevalent in many water bodies, can bind to DNA templates and inhibit PCR amplification. Similarly, fine sediments, particularly those rich in siliceous materials, have been shown to reduce eDNA detection rates. Algal substances may also decrease eDNA detectability, possibly due to the presence of acids, polysaccharides, and phenols that interfere with PCR reactions (20, 21).

To mitigate these challenges, refining eDNA concentration and extraction techniques is essential to help remove potential inhibitors (8, 21), improving the efficiency of subsequent DNA extraction and amplification processes. Additionally, optimizing DNA extraction protocols to include steps that specifically target the removal of inhibitory substances can enhance eDNA purity and yield. For example, the use of certain reagents during extraction has been shown to effectively reduce the impact of inhibitors like humic acids (20, 21, 22).

In addition to water-based sampling, alternative approaches such as soil eDNA analysis (12) could be explored to detect *O.h. quadrasi* in environments where waterborne eDNA may be insufficient. By integrating multiple sampling matrices and refining processing protocols, eDNA detection methods can be further optimized for accurate and consistent results across different ecological conditions.

Nonetheless, eDNA detection offers several advantages over traditional methods. The concordance between the traditional method and the eDNA detection system suggests that the latter may serve as a key environmental surveillance tool, potentially replacing conventional approaches. A key finding was the statistically significant difference and high percent agreement between eDNA detection and traditional snail surveillance methods, further supporting that eDNA technology can reliably complement—or even replace—traditional field methods. eDNA detection allows for the collection of water samples covering a larger area in a shorter time frame, reducing the labor-intensive process of manual snail collection and the need for extensive human resources (3, 7). Additionally, eDNA testing is non-invasive and safer for health authorities and researchers, as it eliminates direct contact with potentially infected snails and exposure to *S. japonicum* during microscopic examination. These factors make eDNA a promising tool for large-scale surveillance, particularly in areas where direct snail observation is difficult or unsafe.

The detection of the snail intermediate host alone is expected, as Ohq colonies are not necessarily infected with Sj. This suggests a potential transmission site, as oval contamination from human or animal feces can complete the parasite’s life cycle. This implies that areas where *O.h. quadrasi* are present should be frequently monitored, and open defecation by humans or animals in these areas should be avoided or prohibited to prevent environmental contamination and the completion of the parasite’s life cycle, thereby mitigating the transmission risk of schistosomiasis.

On the other hand, the detection of *S. japonicum* alone suggests that the sampling point is a contamination site, likely due to defecation by humans or animals. The detection capacity of *S. japonicum* eDNA through qPCR surpasses that of traditional methods (e.g., snail-crushing and microscopy), which are limited to the patent cercarial stage. Since eDNA-based qPCR can detect DNA across the parasite’s life stages in water—including the ovum, miracidium, and cercariae (snail-borne stage) stages (11)—*S. japonicum* eDNA can be identified even in the absence of its snail intermediate host, *O.h. quadrasi*. This capability may indicate not only active transmission sites but also locations of environmental contamination by humans or other mammalian hosts, such as carabaos (23, 24), dogs, cats, and pigs (25).

Consequently, the detection of both target species in a single area suggests an active transmission site where humans and animals may become infected upon contact with water. This underscores the need to prioritize these areas for control efforts and to avoid sourcing water from them until such efforts are successful. This is where mapping the eDNA presence plays a crucial role.

As the eDNA detection system offers a comprehensive approach to identifying target species—particularly in regions where traditional methods may fail to capture small or low-density snail populations (9, 11, 12)—its application to real-time hazard mapping is essential. Mapping the presence of the snail intermediate host, *O.h. quadrasi*, allows local government units and health authorities to identify potential and active transmission sites, enabling targeted vector control. For researchers, knowing the sites where contamination with *S. japonicum* usually occurs can provide insights for further investigation, such as identifying the mammalian species that may primarily contribute to disease transmission through other downstream molecular applications or metagenomics using environmental DNA. Additionally, if eDNA is used consistently in environmental surveillance, we may be able to better understand the temporal changes in the distribution of the parasite and its intermediate host in other endemic areas, as also highlighted in this study.

Although there is no distinct season in schistosomiasis-endemic areas of the Philippines (26), periodic meteorologic change may still impact the snail population, therefore affecting eDNA presence (10, 14). As observed, the presence of *O.h. quadrasi* eDNA differs in each sampling period, with some movements, especially in natural streams, that are notable. These temporal changes in the distribution of *O.h. quadrasi* may significantly affect disease transmission in several ways. The presence of *O.h. quadrasi* may fluctuate due to meteorologic changes. Meteorologic changes also impact environmental conditions such as temperature, water levels, and water quality. According to the collective meteorological data of the Philippine Atmospheric, Geophysical, and Astronomic Services Administration (PAGASA) from 1991 to 2020 (13), the average ambient temperature in Leyte, Philippines, is lower from December through March of the following year, as rainfall increases.

These conditions are favorable for *O.h. quadrasi* (16–18), and because of this, during periods when the intermediate host is more abundant or concentrated in specific areas, the chances of *S. japonicum* transmission may increase. During times of low host abundance, transmission risk may decrease, but pockets of infection may persist in isolated regions, creating a reservoir for future outbreaks when conditions improve for the parasite and its snail intermediate host. As the life cycle of *S. japonicum* is closely tied to the presence and behavior of the snail intermediate host, changes in the snail population, whether due to environmental factors or human activities, could affect the timing and location of the parasite’s transmission. For instance, an increase in snail density—as detected by eDNA—was observed during the December survey. Given that the rice harvest season usually occurs around that time, the increase in human activities, alongside their animal companion, in areas where snail colonies are present may have also increased the chance of environmental contamination with *S. japonicum* ova. The survey conducted in March of the following year may support this, as a significant increase in *S. japonicum* eDNA positivity and co-detection was observed, aligning with the patent stage of *O.h. quadrasi*, which occurs from 8 weeks post-exposure to *S. japonicum* miracidia until the snails’ death (16–18).

Temporal changes in snail distribution can alter where and when humans are exposed to the parasite. Infected snails may shift closer to human activity zones, such as agricultural fields or water sources used for daily activities, especially during flooding. This could result in a higher likelihood of contact with contaminated water during peak seasons of snail activity, increasing the risk of schistosomiasis transmission. Additionally, these shifts can lead to the formation of transmission hotspots, especially if both species co-occur in certain areas during specific periods, as observed in sites 1, 9, 11, and 21. These hotspots would represent high-risk areas for disease transmission, requiring targeted interventions such as snail control, water sanitation efforts, or environmental alteration.

Therefore, consistent environmental monitoring and mapping throughout the year should also be considered, as there are temporal changes in snail distribution, especially during periods with drastic differences in weather—amount of rainfall received—or differences in water level, condition, and quality—as in planting or harvest periods. This is where eDNA detection by qPCR will be useful, as it is rapid and sensitive. This may also provide information as to where safe bodies of water can be used for the livelihood of residents in endemic areas during specific times of the year.

Additionally, the application of eDNA detection as a rapid and more sensitive tool is paramount to real-time hazard mapping, providing valuable insights for public health interventions. The findings of this research highlight the advantages of eDNA detection over traditional methods, particularly in identifying low-density or elusive populations like *O.h. quadrasi* and *S. japonicum*. With its heightened sensitivity, eDNA provides a comprehensive view of species distribution, complementing traditional surveillance methods. Despite occasional non-detections due to environmental factors, refining eDNA protocols will improve their efficacy in complex environments. Moreover, eDNA’s rapid, non-invasive nature and scalability make it an invaluable tool for real-time monitoring, hazard mapping, and understanding disease dynamics. Its integration into public health strategies could enable more effective interventions, especially in areas where human or animal activity heightens the risk of schistosomiasis transmission.

## MATERIALS AND METHODS

### Water sample, snail collection, and examination

Prior to any sampling and/or snail collection, we strictly implemented proper PPE, such as boots and gloves, to ensure the safety of the researchers and assisting village officials. At each sampling site, 500 mL water samples were taken in triplicates (sub-samples) using PET bottles (11). The water samples were collected along the edge of the bodies of water, added 1:500 of 10% wt/vol benzalkonium chloride solution as a preservative, and transported refrigerated in cooler boxes to the laboratory for further processing. At every sampling site, the materials and equipment used for water collection and water-quality testing were rinsed thoroughly before reuse to avoid contamination.

The malacological surveys were conducted at each site by time sampling method (16) for at least 5 minutes. Collected snails were kept in bags labeled with their respective sampling site numbers for temporary storage and transport. In the laboratory, the snails were morphologically identified and microscopically examined for parasites by crushing method (3, 7). Each snail was placed on a glass slide with a drop of water and gently crushed with another glass slide—just enough to break the shell but not crush the snail tissue. The snail tissues were teased apart using a pair of 23-gauge needles to observe whether the furcocercous bifurcated cercariae of *S. japonicum* or a sporocyst was present.

### Environmental DNA saturation using gravity filtration method and Sterivex filter cartridge

After sample collection, the samples were transported and processed immediately at the Research Institute for Tropical Medicine—Palo Satellite Laboratory in Palo, Leyte, located approximately 25 kilometers from Ekiran Village.

Samples were filtered individually by gravity (19), and the eDNA was trapped in cartridge filters (8, 22). Modifications from the methods previous studies were made as follows: the filtration system consisted of a resealable enteral feeding set (Top Cooperation, Tokyo, Japan) attached to a Sterivex filter cartridge (Merck Millipore, Massachusetts, USA) and 2 m of silicon tube. The water sample was poured into the resealable enteral feeding bag and hung at a height of at least 2 meters (19). After all the water had drained, the cartridge filter was changed, and the next aliquot was poured. Each bag was dedicated to a single sampling site to elude contamination and to make it cost-effective. Each filter cartridge was labeled as its corresponding bottle (site and sub-sample numbers).

The eDNA trapped in the cartridge filters was extracted using the QIAGEN DNeasy Blood and Tissue Kit following the pre-processing lysis step employed in the previous eDNA study (8). One milliliter of ATL buffer and 1 mL of AL Buffer-Proteinase k mix (8) (QIAGEN, Hilden, Germany) was added to the cartridge filter and incubated at 56°C for at least 30 minutes. Post-incubation, the filter was attached to a salivette tube (Sarstedt AG & Co. KG, Nümbrecht, Germany) and centrifuged for 3 minutes at 3,000 × *g*. Refrigerated absolute ethanol was added to the lysed filtrate at half the volume collected so that the final concentration of ethanol was one-third of the total volume and incubated in refrigeration for at least an hour, preferably overnight. Afterward, the precipitate was transferred to a spin column (QIAGEN), and the DNA extraction was completed by following the manufacturer’s protocol with a final elution performed twice with 55 µL of elution buffer each time, resulting in a total volume of 110 µL eluate.

### Multiplex qPCR assay for field samples

The samples were analyzed using the primer and probe design described by Fornillos et al. (11). However, to accommodate for a multiplex assay, we changed the primer and probe setup to pre-mixed primer and probe set (Integrated DNA Technologies, Inc., Coralville, IA, USA) and the modification for each species are as follows:

For *O.h. quadrasi*, no modification was made for the reverse primer; however, two additional nucleotides were added to the end of the forward primer, Ohq_COI-F (5′-GCATGTGAGCGGGGCTAGTAGGC-3′), and the probe was changed to its reverse complement and was modified according to the manufacturer's recommendation, Ohq_COI-Probe (5′-[56-FAM]AGGACTGAC[ZEN]CTAACTCTGCAC[3IABkFQ]-3′). For *S. japonicum,* no modification was made to the sequence of both forward and reverse primers; however, the probe design was modified according to the manufacturer's recommendation, Sj_COI-Probe (5′-[5HEX]TTTTGGTAA[ZEN]ATATCTTCTTCCG[3IABkFQ]−3′).

For field applicability, the multiplex assay was performed using a Magnetic Induction Cycler (MIC; Bio Molecular Systems, Upper Coomera, Queensland, Australia) in a 30 µL reaction volume. This reaction volume consisted of 1× TaqMan Environmental Master Mix 2.0 (Applied Biosystems, California, USA), 0.67 pmol of each of the target species’ primer-probe mix (Integrated DNA Technologies, Inc., Coralville, IA, USA), and 5 µL of eDNA sample. The thermal cycling conditions included an initial denaturation at 95°C for 10 minutes, followed by 50 cycles of two-step PCR at 95°C for 15 seconds and 60°C for 60 seconds. A site was considered positive if at least one of the aliquots showed amplification (12). The positive control used for the assay was the genomic DNA from both *O.h. quadrasi* and *S. japonicum*.

### Statistical analysis and hazard mapping

The number of snails collected per site was recorded and compared with the qPCR-*Ohq*-COI-target results while the number of *S. japonicum*-infected *O.h. quadrasi* observed was recorded and compared with the qPCR-*Sj*-COI-target results. The Wilcoxon signed-rank test was used to determine if there is a statistical difference between the traditional method and the eDNA detection system. The Mann-Whitney *U* test was used to determine if there is a significant difference in water quality measures between sites with and without the presence of *S. japonicum* and *O.h. quadrasi* eDNA. *P* values ≤0.05 indicated a statistically significant difference between treatments. Aside from the Wilcoxon signed-rank test statistic, percent agreement (27) was also used to descriptively explain the comparability of the traditional method and the eDNA detection system qPCR.

Maps were generated per sampling period to give an overview of the distribution and movement of *O.h. quadrasi* and *S. japonicum* in the environment over time. The hazard map was created using QGIS version 3.32.3 Lima using the malacological survey, eDNA results, and the GPS data.

## SUPPLEMENTAL MATERIAL

Supplemental material msphere.01061-24-s0001.docx Additional experimental details and Table S1.

ASM does not own the copyrights to Supplemental Material that may be linked to, or accessed through, an article. The authors have granted ASM a non-exclusive, world-wide license to publish the Supplemental Material files. Please contact the corresponding author directly for reuse.

## References

[1] Belizario VJ, de Cadiz AE, Navarro RC, Flores MJC, Molina VB, Dalisay SNM, Medina JRC, Lumangaya CR. 2022. The status of Schistosomiasis japonica control in the Philippines: the need for an integrated approach to address a multidimensional problem. Int J One Health 8:8–19. doi:10.14202/IJOH.2022.8-19

[2] Olveda RM, Gray DJ. 2019. Schistosomiasis in the Philippines: innovative control approach is needed if elimination is the goal. Trop Med Infect Dis 4:66. doi:10.3390/tropicalmed4020066 31013917 PMC6631753

[3] Madsen H, Carabin H, Balolong D, Tallo VL, Olveda R, Yuan M, McGarvey ST. 2008. Prevalence of Schistosoma japonicum infection of Oncomelania quadrasi snail colonies in 50 irrigated and rain-fed villages of Samar Province, the Philippines. Acta Trop 105:235–241. doi:10.1016/j.actatropica.2007.12.002 18207119 PMC2293956

[4] Kamel B, Laidemitt MR, Lu L, Babbitt C, Weinbaum OL, Mkoji GM, Loker ES. 2021. Detecting and identifying Schistosoma infections in snails and aquatic habitats: a systematic review. PLoS Negl Trop Dis 15:e0009175. doi:10.1371/journal.pntd.0009175 33760814 PMC8021170

[5] Department of Health. 2019. 2019-2025 strategic plan towards interruption of sch infection in the Philippines. Manila, Philippines

[6] Hong Z, Li L, Zhang L, Wang Q, Xu J, Li S, Zhou XN. 2022. Elimination of Schistosomiasis japonica in China: from the one health perspective. China CDC Wkly 4:130–134. doi:10.46234/ccdcw2022.024 35265392 PMC8886488

[7] Leonardo L, Chigusa Y, Kikuchi M, Kato-Hayashi N, Kawazu S-I, Angeles JM, Fontanilla IK, Tabios I, Moendeg K, Goto Y, Fornillos RJ, Tamayo PG, Chua J. 2016. Schistosomiasis in the Philippines: challenges and some successes in control. Southeast Asian J Trop Med Public Health 47:651–666.

[8] Wu Q, Minamoto T. 2023. Improvement of recovery yield of macro-organismal environmental DNA from seawater samples. Anal Sci 39:713–720. doi:10.1007/s44211-023-00280-1 36754915 PMC10121502

[9] Sato MO, Rafalimanantsoa A, Ramarokoto C, Rahetilahy AM, Ravoniarimbinina P, Kawai S, Minamoto T, Sato M, Kirinoki M, Rasolofo V, De Calan M, Chigusa Y. 2018. Usefulness of environmental DNA for detecting Schistosoma mansoni occurrence sites in Madagascar. Int J Infect Dis 76:130–136. doi:10.1016/j.ijid.2018.08.018 30201503

[10] Barnes MA, Turner CR. 2016. The ecology of environmental DNA and implications for conservation genetics. Conserv Genet 17:1–17. doi:10.1007/s10592-015-0775-4

[11] Fornillos RJC, Sato MO, Tabios IKB, Sato M, Leonardo LR, Chigusa Y, Minamoto T, Kikuchi M, Legaspi ER, Fontanilla IKC. 2019. Detection of Schistosoma japonicum and Oncomelania hupensis quadrasi environmental DNA and its potential utility to Schistosomiasis japonica surveillance in the Philippines. PLoS One 14:e0224617. doi:10.1371/journal.pone.0224617 31747401 PMC6867693

[12] Calata FIC, Caranguian CZ, Mendoza JEM, Fornillos RJC, Tabios IKB, Fontanilla IKC, Leonardo LR, Sunico LS, Kawai S, Chigusa Y, Kikuchi M, Sato M, Minamoto T, Baoanan ZG, Sato MO. 2019. Analysis of environmental DNA and edaphic factors for the detection of the snail intermediate host Oncomelania hupensis quadrasi. Pathogens 8:160. doi:10.3390/pathogens8040160 31547610 PMC6963648

[13] Philippine Atmospheric, Geophysical and Astronomical Services Administration. 2020. 1991 – 2020 climatological normals of Tacloban City, Leyte. Quezon City, Philippines

[14] Collins RA, Wangensteen OS, O’Gorman EJ, Mariani S, Sims DW, Genner MJ. 2018. Persistence of environmental DNA in marine systems. Commun Biol 1:185. doi:10.1038/s42003-018-0192-6 30417122 PMC6218555

[15] Manalo DL, Bolivar JKG, Yap PR, Gomez MRR, Saldo ZP, Espino MJM, Dilig JE, Fornillos RJC, Perez SA, Baga RA, Sunico LS, Fontanilla IKC, Leonardo LR, Gomez M. 2023. From perpetual wetness to soil chemistry: enumerating environmental and physicochemical factors favoring Oncomelania hupensis quadrasi snail presence in the municipality of Gonzaga, Cagayan, Philippines. Trop Med Infect Dis 9:9. doi:10.3390/tropicalmed9010009 38251207 PMC10819408

[16] Pesigan TP, Hairston NG, Jauregui JJ, Garcia EG, Santos AT, Santos BC, Besa AA. 1958. Studies on Schistosoma japonicum infection in the Philippines. 2. The molluscan host. Bull World Health Org 18:481–578. 13536804 PMC2537606

[17] Leonardo L, Varona G, Fornillos RJ, Manalo D, Tabios IK, Moendeg K, de Cadiz A, Kikuchi M, Chigusa Y, Mistica M, Hernandez L, Palasi W, Fontanilla IK. 2020. Oncomelania hupensis quadrasi: snail intermediate host of Schistosoma japonicum in the Philippines. Acta Trop 210:105547. doi:10.1016/j.actatropica.2020.105547 32479837

[18] Ohmae H, Iwanaga Y, Nara T, Matsuda H, Yasuraoka K. 2003. Biological characteristics and control of intermediate snail host of Schistosoma japonicum. Parasitol Int 52:409–417. doi:10.1016/s1383-5769(03)00058-8 14665400

[19] Oka SI, Miya M, Sado T. 2022. Gravity filtration of environmental DNA: a simple, fast, and power-free method. MethodsX 9:101838. doi:10.1016/j.mex.2022.101838 36117674 PMC9472067

[20] Heid CA, Stevens J, Livak KJ, Williams PM. 1996. Real time quantitative PCR. Genome Res 6:986–994. doi:10.1101/gr.6.10.986 8908518

[21] Stoeckle BC, Beggel S, Cerwenka AF, Motivans E, Kuehn R, Geist J. 2017. A systematic approach to evaluate the influence of environmental conditions on eDNA detection success in aquatic ecosystems. PLoS One 12:e0189119. doi:10.1371/journal.pone.0189119 29220394 PMC5722286

[22] Wong MKS, Nakao M, Hyodo S. 2020. Field application of an improved protocol for environmental DNA extraction, purification, and measurement using Sterivex filter. Sci Rep 10:21531. doi:10.1038/s41598-020-77304-7 33298993 PMC7725969

[23] Wu HW, Qin YF, Chu K, Meng R, Liu Y, McGarvey ST, Olveda R, Acosta L, Ji MJ, Fernandez T, Friedman JF, Kurtis JD. 2010. High prevalence of Schistosoma japonicum infection in water buffaloes in the Philippines assessed by real-time polymerase chain reaction. Am J Trop Med Hyg 82:646–652. doi:10.4269/ajtmh.2010.09-0638 20348514 PMC2844580

[24] Gordon CA, Acosta LP, Gray DJ, Olveda RM, Jarilla B, Gobert GN, Ross AG, McManus DP. 2012. High prevalence of Schistosoma japonicum infection in Carabao from Samar Province, the Philippines: implications for transmission and control. PLoS Negl Trop Dis 6:e1778. doi:10.1371/journal.pntd.0001778 23029571 PMC3447974

[25] Carabin H, Balolong E, Joseph L, McGarvey ST, Johansen MV, Fernandez T, Willingham AL, Olveda R, Schistosomiasis Transmission And Ecology In The Philippines Step Project. 2005. Estimating sensitivity and specificity of a faecal examination method for Schistosoma japonicum infection in cats, dogs, water buffaloes, pigs, and rats in Western Samar and Sorsogon Provinces, The Philippines. Int J Parasitol 35:1517–1524. doi:10.1016/j.ijpara.2005.06.010 16188261

[26] Olveda DU, Li Y, Olveda RM, Lam AK, McManus DP, Chau TNP, Harn DA, Williams GM, Gray DJ, Ross AGP. 2014. Bilharzia in the Philippines: past, present, and future. Int J Infect Dis 18:52–56. doi:10.1016/j.ijid.2013.09.011 24211228

[27] Ranganathan P, Pramesh CS, Aggarwal R. 2017. Common pitfalls in statistical analysis: measures of agreement. Perspect Clin Res 8:187–191. doi:10.4103/picr.PICR_123_17 29109937 PMC5654219

