# Supplementary material for: Seasonality and dynamics of schistosomiasis in the environment: usefulness of environmental DNA (eDNA) surveillance system at a community level for risk mapping schistosomiasis in Ekiran Village, Leyte, Philippines
Source: mSphere. 2025 Mar 26;10(4):e01061-24. doi: 10.1128/msphere.01061-24 (PMC12039233; doi:10.1128/msphere.01061-24)
Supplement: Supplemental material — Additional experimental details and Table S1. [file msphere.01061-24-s0001.docx]

**SUPPLEMENTARY MATERIAL**

Serial dilution experiment for determination of the detection limit of *O.h. quadras*i and *S. japonicum* eDNA-based qPCR assay.

The genomic DNA of a single *S. japonicum* (Sj)-infected *O. h. quadrasi* (Ohq) was extracted using the QIAGEN Blood and Tissue Extraction Kit, following the manufacturer’s protocol. The cytochrome c oxidase I (COI) gene was isolated from both target species through separate amplifications (singleplex qPCR) using the same primer-probe mix applied in this study. The amplicons for both Ohq and Sj were then purified using the FastGene Gel/PCR Extraction Kit to ensure accurate quantification of the COI to be amplified.

The purified COI of both target species was quantified using the NanoDrop Lite Plus (ThermoScientific, Massachusetts, USA). A ten-fold serial dilution was performed, starting from an equated concentration of 5.3 ng/µL for each target species. However, to better simulate field conditions, a multiplex approach was implemented for comparison. The detection limit of the multiplex assay was determined to be 5.3 × 10⁻^11^ ng/µL (1:100 billion dilution). The assay demonstrated the capability to detect eDNA at concentrations as low as 1 copy/5µL.

**Supplementary Table 1.** Serial dilution experiment for determination of the detection limit of *O.h. quadras*i and *S. japonicum* eDNA-based qPCR assay.

| **Assay** | **Dilution** | **DNA Concentration per Species (ng/uL)** | **Sample Name** | ***Ohq COI (Cq)*** | ***Ohq COI***  ***(Copy number/μL)*** | ***Sj COI (Cq)*** | ***Sj COI (Copy number/μL)*** |
| --- | --- | --- | --- | --- | --- | --- | --- |
| **Multiplex**  **Ohq/Sj qPCR** | 1:1 | 5.30 | 1:1 | 4.18 | 26258000823 | 4.71 | 41262572724 |
|  | 1:10 | 5.30E-01 | 1:10 | 5.88 | 2625800082.30 | 6.41 | 4126257272.40 |
|  | 1:100 | 5.30E-02 | 1:100 | 9.59 | 262580008.23 | 10.08 | 412625727.24 |
|  | 1:1000 | 5.30E-03 | 1:1K | 13.67 | 26258000.82 | 14.02 | 41262572.72 |
|  | 1:10000 | 5.30E-04 | 1:10K | 17.83 | 2625800.08 | 18.08 | 4126257.27 |
|  | 1:100000 | 5.30E-05 | 1:100K | 21.72 | 262580.01 | 21.98 | 412625.73 |
|  | 1:1000000 | 5.30E-06 | 1:1M | 25.00 | 26258.00 | 25.04 | 41262.57 |
|  | 1:10000000 | 5.30E-07 | 1:10M | 35.21 | 2625.80 | 35.10 | 4126.26 |
|  | 1:100000000 | 5.30E-08 | 1:100M | 35.57 | 262.58 | 35.44 | 412.63 |
|  | 1:1000000000 | 5.30E-09 | 1:1B | 39.77 | 26.26 | 39.56 | 41.26 |
|  | 1:10000000000 | 5.30E-10 | 1:10B | 43.98 | 2.63 | 43.67 | 4.13 |
|  | 1:100000000000 | 5.30E-11 | 1:100B | 48.19 | 0.26 | 47.79 | 0.41 |
|  | 1: 1000000000000 | 5.30E-12 | 1:1T | ND |  | ND |  |

ND: not detectable
